# Notes of a Case of Nervous Irritation during Pregnancy with Separation of the Symphysis Pulbis

**Published:** 1830

**Authors:** Edwin A. Atlee


### III. Original.

28. *Notes of a case of nervous irritation during pregnancy, with separation of the symphysis pubis.* On the 22d of June 1829, I was sent for, to see Mrs. A—— who was in the 6th month of gestation. In consequence of a slight cold, she had submitted to a sweating process, consisting of copious draughts of stimulating aromatic teas, and of warm applications to the lower extremities; the effects of which were, violent arterial action, threatening congestion in the vessels of the lungs, heart and head, evinced by very hurried respiration, a sense of fulness in the brain, dimness, and syncope.

The pulse was very irregular, and as to frequency, when I first saw her, it was not to be distinctly numbered, but certainly its pulsations exceeded 150 in the miuute. There was no perceptible motion of the fœtus.

I immediately opened a vein in the arm, and detracted blood freely, not desisting until the pulse became evidently much less frequent, and the respiration, which had been very painful, as well as extremely hurried, became more regular and free from pain. Having waited, by her bed side about an hour, and perceived a threatening return of all the symptoms, I bound up the arm, and drew from the orifice, a sufficiency to obviate the danger. I think the quantity taken, by the two venesections, amounted to 24 ounces. In about a quarter of an hour, I left her, promising to return in the course of the day; but before leaving her, I directed about 30 drops of camphorated spirit to be be given, whenever oppressed breathing or faintness came on.

In about 3 hours afterwards, I visited her again, and found her pretty comfortable. She informed me, that a short time before I came in, she perceived the first returning motion of her child, which appeared convulsive, and simultaneous with a very strange sensation about her heart, like three or four strong electric shocks, determining themselves from the heart to the head; after which the motions of the fœtus were as formerly, and, excepting a considerable exhaustion of strength, she had no alarming symptoms.

She however complained, on leaving her bed, of a pain at the symphysis pubis, and considerable difficulty in walking, or standiug. For this I had a bandage applied over the hips, and directed it to be worn constantly.

At the full term of gestation, she brought forth a fine healthy son; but not without completing the separation of the agglutinated bones of the pubis. These however, by long confinement in a horizontal position, and wearing the bandage, 2 months reunited; and she now enjoys good health, and nurses, from full fountains, a thriving urchin.

If time were allowed me, I should add some remarks on this case; but they will naturally present themselves to the reflecting practitioner. My notes of the case, having been mislaid, I have drawn up this hasty paper, from memory.

Such as it is, I respectfully submit it, and am, Sir, with sentiments of high esteem,

Yours,

EDWIN A. ATLEE, M.D.

DR. DRAKE.

Cincinnati, March, 1830.

